# Autologous growth factors used for the treatment of recurrent fistula-in-ano preliminary results

**DOI:** 10.1007/s10151-012-0954-y

**Published:** 2012-11-29

**Authors:** A. Kucharczyk, M. Kołodziejczak, I. Sudoł-Szopińska, K. Bielecki

**Affiliations:** Proctology Unit, Department of General Surgery, Solec Hospital, Warsaw, Poland

Dear Sir,

The risk of postoperative complications such as fistula recurrence or incontinence increases in patients with recurrent fistulas and high transsphincteric and suprasphincteric fistulas [1, 2]. Due to the large number of postoperative complications, in recent years, there have been some attempts to treat this disease conservatively. Methods of treatment include tissue adhesives and plugs that close the internal orifice as well as autologous growth factors that are present in the platelet-rich plasma and used as “natural glue” to close the lumen of the fistula.

**Fig. 1:**
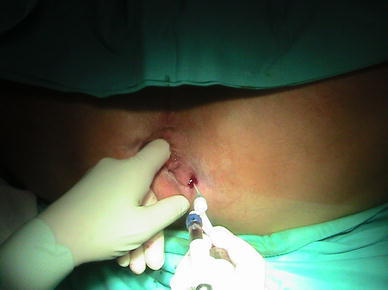
Autologous platelet-rich plasm administration to fistula tract

Autologous platelet-rich plasma (APRP) is platelet concentration derived from centrifuged full blood collected from a patient right before surgery. The platelet-rich plasma (PRP) obtained using the Gravitational Platelet Separation III System (GPS^®^ III, Biomet Merck, UK) constitutes 10 % of the original volume of the collected blood and more than 90 % of its mass is made of platelets. Platelet-rich plasma preparation is administered to the fistula tract following curettage (Fig. 1). We report our experience with the GPS^®^ System in 2 patients with anal fistula.

A 50-year-old male underwent surgery for recurrent suprasphincteric posterior horseshoe fistula at our unit. At a time of surgery curettage of the fistula tract was performed, and then a GPS^®^ III preparation was administered to the fistula tract. The patient was discharged home 2 days after surgery. Fourteen days after surgery, the patient presented at the ward due to an inflammatory infiltration. Two months after surgery, a follow-up examination revealed fistula recurrence.

A 37-year-old male with recurrent intersphincteric anterior fistula-in-ano, and with a narrow, transsphincteric branch running towards the puborectalis loop also underwent surgical treatment at our unit. At a time of surgery, the main tract of the fistula was removed and APRP (GPS^®^ III) was administered to the narrow lateral tract following curettage. The patient was discharged home on postoperative day 3. No recurrence of the fistula was reported within the 6-month follow-up period.

The first experiences with using growth factors were related to the treatment of patients with chronic skin ulcers [3]. At present, indications for using APRP therapy are being expanded. APRP is used in burn units and for the treatment of non-healing wounds. There are also reports of using APRP for the treatment of tendon injuries, muscle injuries, degenerative lesions of tendons and joints and bone voids, mainly in maxillofacial surgery. APRP therapy is also offered by aesthetic surgery centres, where it is used to fill deep wrinkles.

In the healing process, thrombin stimulates platelet alpha-granules to release growth factors which, through appropriate stem cell receptors, stimulate stem cell differentiation.

Reports of using autologous growth factors for the treatment of fistula-in-ano date back to the late 1990s. In 1999, a paper on the treatment of patients with recurrent fistula-in-ano was published [2]. It described a group of 30 patients with complex fistulas, rectovaginal fistulas and vesicorectal fistulas. The cure rate was 60 %. A similar cure rate was reported by Cintron et al. [1].

In the literature, there are reports of using APRP as an element of multi-stage treatment of fistula-in-ano. The first stage involves inserting a loose seton through the fistula tract in order to drain and heal any local inflammatory lesions. The second stage involves APRP administration, which resulted in fistula healing in as many as 75 % of patients [4]. Hagen et al. [5] used the two-stage method, and in 2009 they published the results of administering APRP to 10 patients with high transsphincteric fistulas. The first stage of treatment involved draining the fistula tract and then closing the internal opening with a patch of mucous membrane and filling the tract with APRP. After almost 2 years of follow-up, fistula recurrence was observed in 10 % of the patients.

Available publications include papers that present small groups of patients, different types of fistulas qualified for APRP treatment and different methods of surgery. However, the results are encouraging since this method has no negative effect on anal sphincter function.
